# Editorial: Large Language Models for medical applications

**DOI:** 10.3389/fmed.2025.1625293

**Published:** 2025-05-30

**Authors:** Ariel Soares Teles, Alaa Abd-alrazaq, Thomas F. Heston, Rafat Damseh, Lívia Ruback

**Affiliations:** ^1^Campus Araioses, Federal Institute of Maranhão, Araioses, Maranhão, Brazil; ^2^AI Center for Precision Health, Weill Cornell Medicine - Qatar, Doha, Qatar; ^3^Department of Family Medicine, University of Washington, Seattle, WA, United States; ^4^Computer Science & Software Engineering Department, United Arab Emirates University, Al-Ain, Abu Dhabi, United Arab Emirates; ^5^School of Technology, University of Campinas, Limeira, São Paulo, Brazil

**Keywords:** Large Language Models, LLMs, natural language processing, NLP, medical education, decision support systems

## 1 Introduction

The advent of Large Language Models (LLMs) marks a transformative moment in the evolution of Artificial Intelligence (AI), particularly in their capacity to process and generate human language with remarkable fluency and contextual awareness. These models, trained on vast and diverse corpora, have demonstrated state-of-the-art performance across a range of Natural Language Processing (NLP) tasks. In the medical domain, their potential is especially compelling: from synthesizing complex biomedical literature and supporting clinical decision-making to enhancing patient communication and enabling more equitable access to health information.

This Research Topic was launched to explore the multifaceted role of LLMs in transforming healthcare delivery and medical research. The objective was to gather interdisciplinary contributions that investigate both the capabilities and the limitations of LLMs when applied to clinical decision support, patient engagement, precision medicine, and beyond. We aimed to foster a comprehensive dialogue that includes technical innovations, ethical reflections, and practical case studies. The collection features fifteen published articles, reflecting a diverse range of perspectives and methodological approaches. This Research Topic aspires to illuminate the pathways for integrating LLMs into medical practice while addressing the critical questions that accompany their adoption.

## 2 Clinical decision support and diagnostics

One of the most promising applications of LLMs in medicine lies in their potential to support clinical decision-making and diagnostic reasoning. Ríos-Hoyo et al. assessed GPT-3.5 and GPT-4 on 75 complex diagnostic cases and found that GPT-4 included the correct diagnosis in 68% of cases and ranked it among the top three in 42%. The study highlighted GPT-4's superior accuracy and consistency compared to GPT-3.5, though both models showed limitations. Notably, diagnostic success was more strongly associated with literature prevalence than disease incidence, reinforcing that LLMs should currently be viewed as decision support tools rather than standalone diagnostic systems.

Yin et al. conducted a comparative assessment of four language models, including GPT-4.0, in answering pediatric asthma-related questions. GPT-4.0 showed the highest scores across dimensions such as accuracy and completeness, although all models had limitations in addressing treatment-specific questions. Lee et al. explored the use of GPT-4 as a simulated digital twin for neurological history-taking in cases of headache, stroke, and neurodegenerative disease. Their tripartite model demonstrated 81% overall accuracy in retrieving history of present illness details, supporting the potential of LLMs in structured pre-consultation workflows. Liu et al. presented MED-ChatGPT CoPilot, an AI-assisted system that leverages prompt engineering and GPT-4 to extract structured medical case data from scientific literature, build a vector-based local knowledge base, and deliver diagnostic and therapeutic suggestions through a chatbot interface.

In the oncology domain, Peng et al. developed an interpretable machine learning model for predicting survival in aggressive prostate cancer using SHAP-based explanations. Among nine algorithms tested, LightGBM offered the best prognostic performance, with 1-, 3-, and 5-year AUCs exceeding 0.77. The use of SHAP allowed the identification and ranking of key clinical features influencing survival predictions. Zhang et al. proposed PMPred-AE, a deep learning model based on EfficientNetV2-L with an attention mechanism, for the automatic detection of pathological myopia. The model achieved high accuracy across training, validation, and test sets, and incorporated Grad-CAM for visual interpretability, allowing clinicians to see which retinal regions influenced the model's decisions, making it both an effective and explainable diagnostic tool.

## 3 Patient-facing applications

Studies have showcased how generative AI can directly support patients in managing their health (i.e., a patient-facing LLM application), particularly through accessible and personalized tools. Jin et al. evaluated a GPT-based recipe generation tool designed to improve the nutritional management of individuals undergoing peritoneal dialysis. The pilot study found significant improvements in serum prealbumin levels, suggesting that personalized dietary plans generated via LLMs can be both clinically effective and user-friendly. In another clinical application, Aydin et al. (a) offered a critical perspective on the use of LLMs for patient-centered medication guidance and self-decision support. While highlighting the promise of these tools in enhancing health literacy and supporting patients in remote or resource-limited settings, the authors caution against over-reliance on AI-generated information, particularly in high-stakes scenarios involving drug interactions or complex conditions.

## 4 Education and training

The integration of generative AI into health profession education is prompting both enthusiasm and caution, as emerging research examines its impact on learners' preparedness, skills, and ethical sensibilities. In a reflective opinion article, Sharifi Kelarijani et al. argue that, while tools like ChatGPT offer new opportunities for nursing education (e.g., rapid access to information and assistance with assignments), they also risk diminishing students' critical thinking, communication, and clinical reasoning skills if used without proper pedagogical oversight. Echoing these concerns from a student-centered perspective, Gualda-Gea et al. surveyed senior medical students and found limited prior exposure to AI tools but strong recognition of their future importance. Most students supported integrating AI into the curriculum, though many also expressed concern about ethical implications, potential biases, and over-reliance on chatbot-generated information.

Beyond student attitudes, the practical application of LLMs in health education is beginning to take shape. Aydin et al. (b) conducted a scoping review that selected 201 articles and mapped out how LLMs are currently being used to support patient education, identifying six key themes ranging from generating patient-friendly educational materials to enhancing doctor-patient communication. LLMs were found to demonstrate the ability to deliver accurate answers to patient questions, improve the quality of existing educational content, and rephrase medical information in a way that is easier for patients to understand. Nonetheless, issues related to readability, accuracy, and potential biases remain a concern.

## 5 Medical documentation and synthetic text creation

Two contributions to this Research Topic explored distinct applications of generative AI within the clinical domain. Lu et al. evaluated the use of GPT-4o for generating medical history records and found that its outputs were comparable in quality to those written by resident physicians. This points to a promising role for LLMs in supporting clinical documentation workflows. Differently, Ren et al. addressed challenges related to data access and privacy by proposing a method for generating synthetic clinical letters using pre-trained language models. Their framework enables the creation of de-identified, yet semantically rich, clinical texts that can be used for training and evaluating downstream NLP tasks such as named entity recognition.

## 6 Implications and future directions

LLMs demonstrate promise across varied healthcare contexts but require rigorous evaluation and safeguards. Their integration into medical and healthcare practice by clinicians, nurses, and pharmacists offers the potential to streamline clinical decision support, diagnostics, management, patient-facing applications, education/training, medical documentation, and synthetic text creation. However, realizing this potential demands more than technical advancement; it requires a concerted effort to ensure ethical, transparent, and accountable implementation.

Wang et al. reinforced these considerations through a comprehensive bibliometric analysis of ChatGPT's application in nursing. Their study highlights growing international interest, particularly in domains such as nursing education and clinical decision-making, while also pointing to the fragmented and early-stage nature of the research landscape. Despite increasing publication volume and global engagement, collaboration across author groups remains limited, and ethical concerns, including misinformation, over-reliance, and data security, are insufficiently addressed. These findings underscore the need for interdisciplinary cooperation, empirical evaluation, and a stronger emphasis on responsible innovation as LLMs become more integrated into healthcare practice.

Importantly, as Bélisle-Pipon cautions, we must also reevaluate how we conceptualize the shortcomings of LLMs. Framing their inaccuracies as mere “hallucinations” may obscure the deeper epistemic issue: that these models generate plausible text without any concern for truth. Recasting such failures as “bullshit”, in the philosophical sense of conveying information without regard to accuracy, underscores the serious risks of over-reliance on LLMs in high-stakes clinical settings. This critique invites the medical and healthcare AI community to adopt a more skeptical and reflective posture, one that resists hype and prioritizes verification, contextual understanding, and human oversight.

This Research Topic highlights both the extraordinary potential, ethical aspects, and the current limitations of LLMs in healthcare applications by physicians, nurses, and pharmacists. It offers a foundation for critical inquiry as the field matures. Going forward, the responsible deployment of LLMs in healthcare must be guided not only by innovation but also by ethical foresight, transparency, and a deep commitment to patient well-being.

